# Infection After Hysterectomy

**DOI:** 10.1155/S1064744997000112

**Published:** 1997

**Authors:** David L. Hemsell

**Affiliations:** Department of Obstetrics and Gynecology University of Texas Southwestern Medical Center at Dallas 5323 Harry Hines Boulevard Dallas TX G6.226 USA

## Abstract

Antibiotic prophylaxis and advances in technology have reduced operative site infections after hysterectomy to a minimum. Pelvic infections are the most common infection type and respond promptly to a variety of parenteral single-agent and combination antibiotic regimens. Oral antibiotic regimens following parenteral therapy are unnecessary. Abdominal incision infections are less common than pelvic infections, less common than seromas or hematomas, and usually do not require antimicrobial therapy. Abscesses or infected hematomas require parenteral antimicrobial therapy, and drainage of those located above the cuff will predictably shorten therapy time. With early discharge from the hospital, many infections will not become evident until after the patient is home. For that reason, it is important that the patient's discharge instructions outline symptoms and signs associated with these infections so she can present for care at the earliest possible time.


Infectious Diseases in Obstetrics and Gynecology 5:52–56 (1997)
© 1997 Wiley-Liss, Inc.

### Infection After Hysterectomy

David L. Hemsell*
*Department of Obstetrics and Gynecology, University of Texas Southwestern Medical Center, Parkland Memorial Hospital, Dallas, TX*

### Abstract

**Antibiotic prophylaxis and advances in technology have reduced operative site infections after hysterectomy to a minimum. Pelvic infections are the most common infection type and respond promptly to a variety of parenteral single-agent and combination antibiotic regimens. Oral antibiotic regimens following parenteral therapy are unnecessary. Abdominal incision infections are less common than pelvic infections, less common than seromas or hematomas, and usually do not require antimicrobial therapy. Abscesses or infected hematomas require parenteral antimicrobial therapy, and drainage of those located above the cuff will predictably shorten therapy time. With early discharge from the hospital, many infections will not become evident until after the patient is home. For that reason, it is important that the patient's discharge instructions outline symptoms and signs associated with these infections so she can present for care at the earliest possible time. Infect. Dis. Obstet. Gynecol. 5:52–56, 1997.** © 1997 Wiley-Liss, Inc.

Key words
female pelvic surgery; antibiotics; hysterectomy

The late 1940s and early 1950s brought significant advances to the field of elective pelvic surgery. Surgical skill refinement, adherence to sterile technique, improved suture material, and reduction in lower reproductive tract bacterial inoculum were important advances, but not as important as the introduction of antibiotics. Richards[1] reported in 1944 that the local application of sulfonamide at the time of hysterectomy did not alter infection rates or cause adverse events. Turner[2] reported 6 years later that penicillin vaginal suppositories significantly reduced infectious morbidity following vaginal hysterectomy. Antimicrobial prophylaxis currently is used by most gynecologists performing hysterectomy, irrespective of route. If infection rates are low without prophylaxis, however, antimicrobial prophylaxis is not indicated.

Without prophylaxis, the reported incidence of operative site infection after hysterectomy ranged from 5 to 70%, but most reports were mid-range, and higher after vaginal hysterectomy. It was difficult to identify uniform risk factors for postoperative infection other than lower socioeconomic status, immunocompromised host, and infection or flora overgrowth. A good example of the last variable is the recently reported[3,4] association between preoperative bacterial vaginosis and an increase in the rate of operative site infection following hysterectomy.

Bacteria that have been reported in flora and infection studies and that are potential pathogens recovered from operative site infections after hysterectomy are presented in Table 1. Bacterial inoculum and virulence factors are complex, expensive to elucidate, and difficult to evaluate in the clinical setting. As difficult to identify are cell-mediated and humoral immune system function in each patient. It is ultimately the interaction be-

*Correspondence to: Dr. David L. Hemsell, Department of Obstetrics and Gynecology, University of Texas Southwestern Medical Center at Dallas, 5323 Harry Hines Boulevard, G6.226, Dallas, TX 75235-9032.

Received 2 December 1996
Accepted 14 January 1997

Review Article

**TABLE 1. Potential pathogens recovered from operative site infections**

| Aerobic bacteria | Anaerobic bacteria |
|---|---|
| Gram-positive cocci | Gram-positive cocci |
| *Enterococcus faecalis* | *Peptostreptococcus* sp. |
| *Streptococcus agalactiae* | |
| *Streptococcus* sp. | Gram-negative bacilli |
| *Staphylococcus aureus* | *Prevotella bivius* |
| *Staphylococcus epidermidis* | *Prevotella* sp. |
| | *Bacteroides fragilis* group |
| *Gram-negative bacilli* | *B. caccae* |
| *Acinetobacter* sp. | *B. distasonis* |
| *Citrobacter* sp. | *B. melaninogenicus* |
| *Enterobacter* sp. | *B. ovatus* |
| *Escherichia coli* | *B. thetaiotaomicron* |
| *Haemophilus influenzae* | *B. uniformis* |
| *Klebsiella* sp. | *Fusobacterium* sp. |
| *Proteus* sp. | |
| *Pseudomonas* sp. | Gram-positive bacilli |
| | *Clostridium* sp. |
| | *Bifidobacterium* sp. |
| | *Eubacterium sp.* |
| | *Lactobacillus* sp. |
| | *Propionibacterium* sp. |
| | |
| | Gram-negative cocci |
| | *Veillonella parvula* |

tween these variables, with modifications by mechanical or antimicrobial applications, which results in the development of a clinically important operative site infection.

Hysterectomy has been classified as a "clean contaminated" procedure because of the lower reproductive tract flora and its introduction into the operative site at surgery. There is little risk of contamination of pelvic tissues until vaginal transection. That the entire procedure is carried out in a contaminated field would explain why reported infection rates following vaginal hysterectomy were higher than those after abdominal hysterectomy, and why antimicrobial prophylaxis was more predictably effective in preventing operative site infection in women undergoing vaginal hysterectomy.

The following is one classification for the types of infection that can develop following hysterectomy. Temperature elevation alone is not the hallmark of operative site infection requiring antimicrobial therapy. *Vaginal cuff cellulitis* undoubtedly happens after every hysterectomy, irrespective of surgical approach. The vaginal surgical margin is erythematous, edematous, and hyperemic early following surgery, and there are purulent vaginal secretions. This occurs with or without prophylaxis, and is not usually, but may be associated with temperature elevation and/or symptoms. If unassociated with symptoms and signs of infection, it does not require treatment and disappears spontaneously. This physiologic response to incision may or may not contribute to recurring temperature elevation in an asymptomatic patient, *febrile morbidity*, which usually occurs during the first 48 h after surgery. If symptoms develop, they are primarily central, lower abdominal and pelvic discomfort, and there is tenderness at the vaginal surgical margin on bimanual examination without evidence of parametrial involvement. This may not be clinically evident until after the patient is discharged from the hospital.

*Pelvic cellulitis*, or lateral extension of cuff cellulitis, is the result of the inability of physician and host to control the inflammatory response to the vaginal surgical margin. Inflammation spreads laterally to the parametrial region(s), and is associated with lower abdominal, pelvic, back, and/or leg pain and recurring temperature elevation, usually ≥38°C, which begins 48–72 h after surgery. At examination, there is lower abdominal tenderness, which is usually more pronounced on one side. No distinct masses are palpable at bimanual examination, although pelvic tissue tenderness is obvious and corresponds to tender area(s) discovered at abdominal examination.

If a tender mass is palpable in the operative site of a woman with posthysterectomy pelvic infection, there are several possibilities. The patient may have a *phlegmon*, an area of intense cellulitis and microabscesses. This tender, indurated area is usually not diagnosed by sonography, but may be identified by a pelvic computerized tomographic (CT) scan, or magnetic resonance imaging. A *pelvic abscess* also may complicate cellulitis. The most frequent location for this more fluctuant tender mass is just above the vaginal surgical margin; it is frequently referred to as a *cuff abscess*. Vaginal probe sonography is more sensitive than transabdominal sonography for identifying such an abscess. An abscess may also develop laterally in the extraperitoneal space or in retained adnexa. If the patient has an infected hematoma or abscess that is above the vaginal surgical margin, draining that site by opening the vaginal cuff will facilitate cure. This can usually be done in a treatment room and does not require another operative procedure. If abscesses are in the lateral pelvic tissues and inaccessible to drainage from the cuff, percutaneous CT-guided drainage may be necessary, but usually is not. There rarely may be a requirement for laparotomy. Rupture of an abscess in(to) the peritoneal cavity is an event that requires immediate surgical therapy.

Many abdominal incision margins normally may have a minimal amount of erythema while healing. If the patient develops wound pain with advancing erythema, fever, and tenderness, *wound infection* is present. These typically develop 4 or 5 days after surgery. Purulent exudate is usually found in varying volumes; it is also possible to have a positive wound culture in the absence of purulent material in an infected wound. Given time, purulent secretions would be present. Seromas and hematomas are not included in the infection category.

The patient may develop an operative site hematoma which is asymptomatic, but which serves as an excellent growth medium for inoculated flora. This may become an *infected hematoma,* and can be located either centrally or laterally in the extraperitoneal spaces or in an abdominal incision. A hematoma is not usually associated with symptoms initially, and is neither palpable nor tender. The only indication of its presence is an unexplained drop in hemoglobin. Even if temperature elevation develops, patients are usually asymptomatic and the hematoma is not palpable in the pelvis at bimanual examination, but vaginal probe sonography is an effective means for detection.

Temperature elevation is the first sign of infected hematoma, and does not occur until the 4th postoperative day or later, now a time when patients are at home. Without treatment, and after several days of fever, pain develops and the infected hematoma is detectable at bimanual examination as a tender mass. It is important therefore to diagnose a hematoma before the patient is discharged. Parenteral antibiotic therapy is indicated for an infected hematoma. Studies have not been performed to determine whether oral antibiotic would prevent clinical infection in an uninfected hematoma. When located in an abdominal incision, a hematoma is more clinically obvious and accessible and is therefore diagnosed and treated (evacuation) before infection develops.

Wound infection definitions were recently reviewed by the Centers for Disease Control and Prevention (CDC)[5] and constitute another and more general set of definitions. In their classification, surgical site infections (SSI) are divided into 1) organ or space infection and 2) incisional infections.

**TABLE 2. Specific organ/space SSI**

| | |
|---|---|
| Sinusitis | Breast abscess or mastitis |
| Disc space | Arterial or venous infection |
| Vaginal cuff | Eye, other than conjunctivitis |
| Ear, mastoid | Spinal abscess without meningitis |
| Endometritis | Intracranial, brain abscess, or dura |
| Endocarditis | Upper respiratory tract, pharyngitis |
| Mediastinitis | Other infections of the urinary tract |
| Osteomyelitis | Oral cavity (mouth, tongue, or gums) |
| Joint or bursa | Other male or female reproductive tract |
| Gastrointestinal tract | Intra-abdominal, not specified elsewhere |
| Meningitis or ventriculitis | Other infections of the lower respiratory tract |
| Myocarditis or pericarditis | |

*Organ or space infections* are defined as those that develop in surgical sites other than the abdominal incision that was opened during the operative procedure. An example would be pelvic infection after hysterectomy. Table 2 lists the specific organ/space surgical infection sites included in their definitions. These infections must occur within 30 days of the hysterectomy and must be accompanied by at least one of the following: 1) purulent drainage from a drain placed through a stab wound into the organ/space; 2) organisms isolated from an aseptically obtained culture of fluid or tissue in the organ/space; 3) an abscess or other evidence of infection involving the organ/space identified during reoperation or by radiologic or histopathologic examination; or 4) diagnosis of a SSI by a surgeon or, attending physician.

Because of the difficulty in the ability to aseptically obtain a culture from a pelvic infection site, most infections are diagnosed clinically based on patient symptoms, vital signs, and physical findings at examination. Radiologic examination may be useful in diagnosing an infected hematoma or an abscess, however. Urine culture, complete blood count (CBC) with differential count, blood cultures, and chest X-ray are seldom necessary, but hemoglobin should be monitored and an unexplained decrease should be investigated.

It must be remembered that a patient may also develop non-surgical site infections after elective pelvic surgery. For that reason, a complete examination designed to detect an infection in any location must be performed when evaluating the patient for operative site infection after hysterectomy.

*Incisional surgical site infections* are subdivided in the CDC definitions into superficial and deep infections. Superficial infection must occur within 30 days and involve only the skin or skin and subcutaneous tissues. Either organisms isolated from an aseptically obtained culture, or purulent drainage must be present in the incision. One of these also must be accompanied by at least one of the following symptoms or signs: 1) redness or heat; 2) localized swelling; 3) pain or tenderness; 4) deliberate opening of incision by a surgeon (unless culture negative); or 5) diagnosis by the surgeon or attending physician. These definitions do not include stitch abscesses.

*Deep incisional surgical site infections* must occur within 30 days after hysterectomy. The diagnosis is confirmed by at least one of the following: 1) purulent drainage from a deep incision, but not the organ or space component of the surgical site; or 2) a deep incision that is deliberately opened by a surgeon when the patient has at least one of the following signs or symptoms: 1) temperature >38°C; 2) an abscess or other evidence of infection found at reoperation, by radiologic examination, or at reoperation by histopathologic diagnosis; or 3) if the diagnosis is made by the surgeon or attending physician. This diagnosis can also be made if a deep incision dehisces spontaneously.

When symptomatic cuff cellulitis develops, the non-penicillin allergic patient often can be treated with an oral broad spectrum regimen such as amoxicillin with sodium clavulanate (Augmentin® 250–500 mg every 8 h. Clindamycin 450 mg orally every 6 h is an alternative for the penicillin allergic patient. For most women with pelvic infections other than cuff cellulitis, parenteral antibiotic therapy is necessary, and women usually promptly respond to parenteral antimicrobial therapy. Parenteral antibiotics should not be administered to women given the diagnosis of *presumed* pelvic infection without a pelvic examination.

Table 3 lists alphabetically several tested and reported regimens that have been effective in the treatment of women who develop operative site pelvic infections after hysterectomy. Parenteral therapy is continued until the patient has been afebrile 24–36 h. Oral antibiotic therapy is unnecessary after successful parenteral therapy.[6]

**TABLE 3. Tested and reported regimens that have been effective in the treatment of women with operative site infections after hysterectomy**

| |
|---|
| Ampicillin/sulbactam (Unasyn®) 3 g q 6 h |
| Cefotaxime (Claforan®) 1 g q 8 h |
| Cefotetan (Cefotan®) 2 g q 12 h |
| Cefoxitin (Mefoxin®) 2 g q 6 h |
| Imipenem/cilastatin (Primaxin®) 500 mg q 8 h |
| Meropenem (Merem®) 500 mg q 8 h |
| Piperacillin (Piperacil®) 4 g q 6 h |
| Piperacillin and tazobactam (Zosyn®) 3.375 g q 6 h |

The most important consideration in antibiotic selection is predictable efficacy. Infections are polymicrobial, and a broad spectrum of antibacterial activity is necessary. The regimens listed in Table 3 have comparable clinical efficacy in reported trials. If regimens are comparable in efficacy, then safety profile considerations are important. If efficacy and safety profiles are similar, then cost considerations become important. Without doubt, combination antimicrobial therapy is also effective in the treatment of women with operative site infection following hysterectomy. No comparative trials have shown combination therapy to be significantly more effective than single-agent therapy, however, and multiple antibiotic regimens are usually more expensive and always more labor intensive for nurses. Clindamycin resistance is increasingly reported, and we have observed an increasing percentage of operative site infections that were not cured until metronidazole was substituted for clindamycin.

Patients that have a true type I hypersensitivity reaction to penicillin antibiotics should not be administered penicillins, cephalosporins, penems, etc. Women who do not have a type I hypersensitivity reaction to penicillin but develop a rash, etc., can be administered a cephalosporin. If a patient has a type I hypersensitivity reaction to penicillin, then therapy should be combination and will contain an anti-anaerobic antibiotic such as clindamycin, 900 mg every 8 h or metronidazole at a loading dose of 15 mg/kg, and then a maintenance dose of 7.5 mg/kg every 6 h. Either should be combined with an aminoglycoside, an antibiotic family effective against the gram-negative aerobic bacteria. Gentamicin appears to be as effective as other aminoglycosides in the treatment of women with posthysterectomy pelvic infection, and is the least expensive.

Although gentamicin was historically administered parenterally every 8 h, recent data indicate that either 5 or 7 mg/kg once daily is as effective, less expensive, and even less toxic than gentamicin administered 3 times daily[7–9] to patients with normal renal function. Peak and trough testing is unnecessary with once daily dosing. If there is no penicillin allergy, ampicillin 2 g every 6 h to cover gram-positive aerobic bacteria should be added to the metronidazole/gentamicin regimen, but may not be necessary for women treated with clindamycin and gentamicin. Vancomycin at a dose of 500 mg every 6 h should be given to women with a type I hypersensitivity reaction to penicillin in place of ampicillin. Newer quinolone antibiotics currently undergoing phase III clinical trials have an exceptionally broad spectrum of activity, and will be available in parenteral and oral preparations.

During treatment for infection, the patient should be monitored at least twice daily to insure success and to detect any adverse reaction which might develop. Almost all superficial incisional infections respond to opening and draining the area with local wound care 3 times daily consisting of irrigation and debriding with solutions such as hydrogen peroxide or acetic acid followed by normal saline. Fine mesh gauze applied directly to the incisional margins and medial packing with 4 × 4s, or other sterile packing speeds granulation tissue development. Antibiotic therapy is not usually necessary. These wounds may be closed or allowed to heal by secondary intention.

Antibiotic treatment failures are uncommon. They may result from 1) development of resistant species; 2) superinfection by different species in the infection site; 3) lack of coverage for the pathogen by the empiric regimen; or 4) volume of infection. Adding to or changing the empiric antibiotic regimen to expand predictable coverage will be successful. In patients with an infected hematoma or abscess, draining will usually facilitate cure. The patient also may have a non-operative site infection, and drug fever must be remembered.

### REFERENCES

1. Richards WR: An evaluation of the local use of sulfonamide drugs in certain gynecological operations. Am J Obstet Gynecol 46:541–545, 1944.
2. Turner SJ: The effect of penicillin vaginal suppositories on morbidity in vaginal hysterectomy and on the vaginal flora. Am J Obstet Gynecol 60:806–812, 1950.
3. Soper DE, Bump RC, Hurt WG: Bacterial vaginosis and trichomoniasis vaginitis are risk factors for cuff cellulitis after abdominal hysterectomy. Am J Obstet Gynecol 163:1016–1023, 1990.
4. Larson P-G, Platz-Christensen J-J, Forsum U, Pahlson C: Clue cells in predicting infections after abdominal hysterectomy. Obstet Gynecol 77:450–452, 1991.
5. Horan TC, Gaynes WP, Martone WJ, Jarvis WR, Emori TG: CDC definitions of nosocomial surgical site infections, 1992: A modification of CDC definitions of surgical wound infections. Infect Control Hosp Epidemiol 13:606–608, 1992.
6. Hager WD, Pascuzzi M, Vernon M: Efficacy of oral antibiotics following parenteral antibiotics for serious infections in obstetrics and gynecology. Obstet Gynecol 73:326–329, 1989.
7. Tulkens PM, Clerckx-Braun F, Donnez J, et al.: Safety and efficacy of aminoglycosides once-a-day: Experimental data and randomized, controlled evaluation in patients suffering from pelvic inflammatory disease. J Drug Dev Suppl 3:71–82, 1988.
8. Belliveau PP, Nicolau DP, Nightingale CH, Quintiliani R: Once-daily gentamicin: Experience in one hundred eighteen patients with postpartum endometritis. J Infect Dis 1:11–18, 1995.
9. del Priore G, Jackson-Stone M, Shim EK, Garfinkel J, Eighmann MA, Frederiksen MC: A comparison of once-daily and 8-hour gentamicin dosing in the treatment of postpartum endometritis. Obstet Gynecol 87:994–1000, 1996.
